# Evaluate of heavy metal concentration in shrimp *(Penaeus semisulcatus*) and crab (*Portunus pelagicus*) with INAA method

**DOI:** 10.1186/2193-1801-2-72

**Published:** 2013-02-28

**Authors:** Marzieh Heidarieh, Mohammad Ghannadi Maragheh, Mehrdad Azizi Shamami, Mehdi Behgar, Farhood Ziaei, Zahra Akbari

**Affiliations:** 1 Agricultural, Medical and Industrial Research School (AMIRS-NSTRI), Karaj, Iran; 2 Nuclear Science and Technology Research Institute (NSTRI), Tehran, Iran

**Keywords:** Heavy metal, Crab, Shrimp, Persian gulf, Neutron activation

## Abstract

The level of the heavy metal in green tiger shrimp (*Penaeus semisulcatus*) and crab (*Portunus pelagicus*) caught off the Persian Gulf near Bushehr province were investigated. This study was performed to evaluate instrumental neutron activation analysis (INAA) to analyze heavy metal concentration in crab and shrimp whole body tissue. The order of the swimmer crab and shrimp heavy metal concentrations were Zn>Fe>As>Mn>Co and Fe>Zn>Mn>As>Co, respectively. The results showed swimmer crab (*Portunus pelagicus*) and shrimp (*Penaeus semisulcatus*) caught off Persian gulf, were contaminated with high level of As (21.38±3.31ppm and 8.28±2.82 ppm, respectively). High levels of As and Mn were noted in crabs and shrimp, respectively.

## Introduction

Heavy metal pollutions are particularly hazardous contaminants in food and the environment. In general, they are not biodegradable and have long biological half-lives. According to the World Health Organization (World Health Organization 1995) heavy metals must be controlled in food sources in order to assure public safety. Excessive concentration of food heavy metals is associated with the etiology of a number of diseases, especially cardiovascular, renal, neurological, and bone diseases (Chailapakul et al. 2008). A major reason to monitor levels of toxic metals in foods follows from the fact that contamination of the general environment has increased.

It is known that some shrimp and crab may provide useful means of monitoring such elemental concentration levels and their impact on the aquatic environment. In 2005BU-Olayan and Thomas showed higher trace metal levels in benthic molluscs and annelids of Kuwait Bay in the Persian Gulf compared to other regions of the word. Al-Mohanna and Subrahmanyam (2001) demonstrated Zn and Cu pollution swimmer crabs and attributed this to the 1991 Gulf War oil spill into Kuwait’s marine environment. Alyahya et al. (2011) reported that concentrations of Cd, Pb, Cu, and Zn in fresh parts of the clam (M. meretrix) in the Persian Gulf near the Saudi Arabia coast line were within the acceptable standard range. To the extent of the author's knowledge, few studies reported heavy metals pollution of shrimp and fish in the Persian Gulf water of Iran (Pourang et al. 2005; Raissy et al. 2011).

Detection methods of stable elements included colorimetry, spectrography, mass spectroscopy, atomic absorption, spectrophotometry, and neutron activation analysis (NAA). Of these methods NAA has advantages over alternative methods. Detection sensitivity are greatest (<0.01 ppm) with neutron activation analysis (NAA) (Corliss 1963) and NAA also allows for simultaneous detection of many other metals in a sample. There is no need to sample preparation (i.e. dry ashing or wet ashing) before the analysis and intact samples can be used for analysis. Instrumental neutron activation analysis (INAA) has been successfully used to investigate heavy metals of many biological materials such as food and freshwater fish (Cunningham and Stroube 1987; Ndiokwere 1983) and also used to determine the body composition of salmon (Talbot et al. 1986).

This study is aimed in determining the concentrations of the heavy metal contamination (Mn, Fe, Zn, Co and As) in green tiger shrimp (*Penaeus semisulcatus*) and blue crab (*Portunus pelagicus*) by using instrumental neutron activation analysis (INAA).

## Material and methods

### Collection and preparation of samples

**Figure 1:**
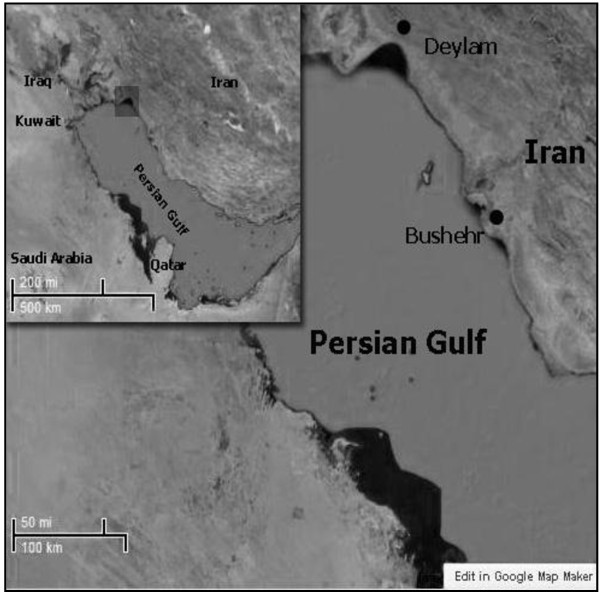
**Location map of sampling site, Deylam port, near Bushehr province area of Iran.**

Swimmer crab (Portunus pelagicus) and shrimp (Penaeus semisulcatus) were captured in depth 41–42 m - Deylam port (Figure 1) by using cast nets during spring of 2011. At this study used 10 same sample size, shrimp (10–12 g) and Crab (11–13 g). Immediately, after sampling crab and shrimp were stored in a container, preserved in crushed ice and transferred to the laboratory and frozen -20°C until analyzed.

Samples (whole body) were dried (60°C for 72 h) and ground through a 2 mm screen for subsequent INAA. The 500–600 mg of dried samples and standard (Fish Tissue, IAEA-407) were accurately weighed into polyethylene vials and heat sealed for irradiation.

### Irradiation and counting

The irradiation of the samples and standard were carried out in the 40-tube specimen rack of the Tehran Research Reactor (Pool Type Reactor) at a neutron flux of 1.0 × 10^13^ n/cm/s. Each analytical portion was irradiated twice, once to analyze for elements yielding short-lived radioisotopes and a second time to analyze for elements yielding long-lived radioisotopes. Table 1 lists the radioisotopes, half-lives and gamma-ray lines used.

**Table 1:** **Radioisotopes, half-lives and gamma-ray energies used**

Isotope	Half-life	Gamma-ray energies (keV)
^56^Mn	155 min	846.76, 1810.72
^59^Fe	44.5 d	1099.25, 1291.60
^60^Co	5.27 y	1173.2, 1332.50
^76^As	1.10 d	559.10
^65^Zn	243.9 d	1115.55
^69m^Zn	825.6 min	438.63
^71m^Zn	236.4 min	386.28

For short-lived radioisotopes, samples were irradiated for 10 seconds and counted 4 minutes later for 300 seconds and again 2 hours later for 1000 seconds. For long-lived radioisotopes, samples were irradiated for 1 hour (3600 s) and counted 3 days later for 1000 seconds and also 10 days later for 3000 seconds and 24 days later for 5000 seconds.

The samples and the standard were counted on the high pure germanium detector (resolution 1332.5 keV at 2.0 keV and efficiency of 20%).

### Statistical analysis

The results were subjected to emulate and analysis using Maestro II and SPAN, respectively.

Animal Dead tissues handling was performed with regard to Iranian animal ethics society and local university rules.

## Results and discussion

The Mn, Fe, Zn, Co and As levels were determined in the whole body of the swimmer crab and green tiger shrimp (Table 2). There are remarkable differences in the swimmer crab and green tiger shrimp heavy metal concentrations. The order of the heavy metal concentrations in swimmer crab was found: Zn>Fe>As>Mn>Co, while in whole body of green tiger shrimp was Fe> Zn>Mn>As>Co.

**Table 2:** **Mn, Fe, Zn, Co and As concentrations (means ± s) in crab and shrimp samples (ppm/dry whole body)**

Metal	Crab	Shrimp
Mn	1.91 ± 0.33	25.43 ± 2.95
As	21.38 ± 3.31	8.28 ± 2.82
Co	0.15 ± 0.02	0.40 ± 0.05
Fe	62.87 ± 11.07	288 ± 38.88
Zn	66.64 ± 7.60	68.73 ± 7.84

The swimmer crab heavy metal concentrations were less than shrimp samples, with the exception of As. The As concentration were 18.07-24.69 and 5.46-11.10 ppm in whole body of crab and shrimp, respectively.

Pourang et al. (2005) examined green tiger shrimp heavy metal distribution in the northwestern (near the Bushehr Province coastline) part of the Persian Gulf. In this study highest mean of Zn concentration (43.39 ppm/fresh weight) was found in hepatopancreas. Also, the Zn levels of the exoskeleton and muscle were 8.56 and 8.98 ppm/wet weight, respectively. In the current study, the level of the Zn/fresh weight of whole body was 22.76 ppm.

Research on swimmer crab in turkey showed Zn, Mn and Fe levels of swimmer crab were 37.2-46.8, 1.30-1.9 and 4.5-6.8 ppm of dry body meat, respectively (Gökoğlu and Yerlikaya 2003). Also, Gökoğlu and Yerlikaya (2003) showed the Fe level of the swimmer crab was higher than results found in the current study.

Sadiq et al. (1982) reported levels of Zn and Co are 165.73 and 4.66 ppm/dry crab whole body, respectively. These values are markedly higher than levels obtained in the current study (66.64 and 0.16 ppm, respectively).

Ayas and Özoğul (2011) showed the concentration of the Zn and Fe were 101.40-104.13 and 21.92-23.90 ppm for male, and 106.13-108.64 and 13.15-16.53 ppm for female swimmer crab, respectively.

The mean values of the heavy metals contents in edible tissue and whole body of crab (*Portunus pelagicus*) and shrimp (*Penaeus semisulcatus*) have been summarized at Table 3. Data obtained from current study indicated that heavy metal contents in crab and shrimp are comparable to the other parts of Persian Gulf and world areas. According to available literature, there is no study on Mn concentration in shrimp tissue.

**Table 3:** **Comparison of mean concentrations of trace metals reported for shrimp (Penaeus semisulcatus) and crab (Portunus pelagicus) in the worldwide**

Location	Tissue	Mn	Fe	Zn	As	Co	Reference
**Crab**							
Persian Gulf, Iran	Whole body	1.91	62.87	66.64	21.38	0.15	Present study
Persian Gulf, Kuwait	Muscle	0.95	-	206.0	0.31	-	Al-Mohanna and Subrahmanyam 2001
Mersin bay, Turkey	Muscle	-	18.93	104.82	-	-	Ayas and Özoğul 2011
Persian Gulf, Saudi Arabia*	Whole body	-	-	165.73	-	4.66	Sadiq et al. 1982
**Shrimp**							
Persian Gulf, Iran	Whole body	25.43	288.0	68.73	8.28	0.40	Present study
Iskenderun bay, Turkey ^1^	Muscle	-	18.69	27.75	-	-	Firat et al. 2008
Persian Gulf, Iran*	Muscle	-	-	41.76	-	-	Pourang et al. 2005
Persian Gulf, Saudi Arabia*	Whole body	-	-	148.89	-	4.56	Sadiq et al. 1982

Values are expressed in ppm per dry weight either of edible tissue or whole body.

1 Mean of males and females.

2 Mean of four stations.

* The values calculated based on dry matter content of these species from the metal concentration per wet weight of shrimp and crab.

Although, in the current study was measured Zn and Mn concentration 56.39-221.68 and 0.14-2.01 ppm, respectively.

Conversely, in study by Al-Mohanna and Subrahmanyam (2001), determined maximum values for As was about 36 times higher than the value was reported.

In most studies the heavy metal concentration are reported either in various body parts of crustaceans or in their edible tissue. However in current study whole body samples were used. Firat et al. (2008) reported higher concentration of heavy metals in hepatopancreas compared to the gill and muscle of shrimp, similar results were reported by Pourang et al. (2005). Hence the heavy metal concentrations of muscle in shrimp and crab samples in the current study might be lower than the reported values.

Sadiq et al. (1982) reported Zn and Co concentrations in shrimp as 148 ppm and 4.56 ppm, respectively. The average Co content was determined as 0.15 ppm and 0.40 ppm in the swimmer crab and shrimp, respectively.

Level of As in the whole body of swimmer crab in the current study was higher than previous studies that reported As concentration in lobster, bivalves, crabs and fishes of Persian gulf (Mora et al. 2004; Al-Mohanna and Subrahmanyam 2001; Raissy et al. 2011). Levels of As contamination in the Persian Gulf may have affected these values. Major anthropogenic sources of arsenic include mining and smelting operations, emissions from coal burning electrical generating facilities, leaching from hazardous waste facilities and from insecticide, herbicide or algicide applications (Anonymous 2009).

Among these sources, herbicide and algicide may have major importance. In recent years local use of algicide against algae bloom has been increased. However, the majority of the As in shrimp and crab appears to be in the form of the less toxic organic form, for example the predominant form is arsenobetaine (Shiomi et al. 1984; Sloth et al. 2005). For this reason, the determination of the total amount of the arsenic in a sample is not sufficient to assess the risk from eating seafood, and speciation analysis is necessary.

High levels of Mn in shrimp samples were noted when compared to crab samples (25.43 ppm vs 1.91 ppm). In a geochemistry study of sediment core of Persian gulf near Bushehr port not only Mn level among selected trace metals was highest, but also the heavy metals concentration were higher than mean crust and mean world sediments (Karbassi et al. 2005; Fazaeli 2010). With the exception of the As, levels of other heavy metals were higher in shrimp compared to crabs. In contrast to these results Sadiq et al. (Sadiq et al. 1982) showed higher heavy metals accumulation in crabs compared to shrimp.

The results showed swimmer crab (*Portunus pelagicus*) and shrimp (*Penaeus semisulcatus*) have been contaminated with heavy metals. Shrimp showed a higher potential to accumulate metals in their body compared to crabs. Further studies are necessary to evaluate the effect of organs, sex, size, season and site of sampling on heavy metal concentration in crab and shrimp. More speciation analysis is also necessary to determination of Pb, Hg, total and organic forms of arsenic.
